# Identification and characterization of alternative splicing variants of buffalo LXR*α* expressed in mammary gland

**DOI:** 10.1038/s41598-022-14771-0

**Published:** 2022-06-22

**Authors:** Xinyang Fan, Yongyun Zhang, Lihua Qiu, Wei Zhu, Xingtiao Tu, Yongwang Miao

**Affiliations:** 1 Faculty of Animal Science and Technology, Yunnan Agricultural University, Kunming, 650201 Yunnan China; 2 Teaching Demonstration Center of the Basic Experiments of Agricultural Majors, Yunnan Agricultural University, Kunming, 650201 Yunnan China; 3 College of Veterinary Medicine, Yunnan Agricultural University, Kunming, 650201 Yunnan China

**Keywords:** Transcription, Gene expression, Gene regulation

## Abstract

Liver X receptor *α* (LXR*α*) is a ligand-dependent transcription factor and plays an important role in the regulation of cholesterol homeostasis, fatty acid biosynthesis and glucose metabolism. In this study, transcripts of *LXRα* gene were cloned and characterized from buffalo mammary gland, and three alternative splicing transcripts of buffalo *LXRα* gene were identified, named *LXRα1*, *LXRα*2 and *LXRα*3. The structure of the *LXRα* transcripts of buffalo and cattle was highly similar. Bioinformatics analysis showed that LXR*α*1 contains two complete functional domains of LXR*α*, one is the DNA-binding domain (NR_DBD_LXR) and the other is the ligand-binding domain (NR_LBD_LXR). The reading frame of *LXRα2* is altered due to the skipping of exon 9, which truncates its encoding protein prematurely at the 400th amino acid residue, making it contain a complete DNA-binding domain and part of a ligand-binding domain. Due to the deletion of exon 4, the protein encoded by *LXRα3* lacks 89 amino acid residues and contains only a complete ligand-binding domain, which makes it lose its transcriptional regulation function. In addition, motifs and conserved domains of three LXR*α* variants of buffalo were highly consistent with those of corresponding transcripts from other mammal species. Subcellular localization analysis showed that LXR*α*1 plays a functional role in the nucleus of buffalo mammary epithelial cells, while LXR*α*2 and LXR*α*3 are distributed in the nucleus and cytoplasm. Compared with non-lactating period, the mRNA abundance of the three *LXRα* transcripts in the mammary gland tissue of buffalo increased during lactating period, revealing that they play a key role in the synthesis of buffalo milk fat. Among the three LXR*α* transcripts, *LXRα1* has the highest expression in the mammary gland, indicating that it is the major transcript in the mammary gland and has important regulatory functions, while LXR*α*2 and LXR*α*3 may have regulatory effects on the function of LXR*α*1. This study highlights the key role of LXR*α* alternative splicing in the post-transcriptional regulation of buffalo lactation.

## Introduction

Nuclear receptors are composed of ligand-activated transcription factor superfamily, which can convert hormone signals into transcriptional responses^1^. The liver X receptor *α* (LXR*α*), as one of the main member of this superfamily, is an oxysterol-inducible transcription factor which can regulate the expression of genes involved in cholesterol and fatty acid metabolism, cellular proliferation and apoptosis, and immunity^2,3^. LXR*α* and retinoid X receptor (RXR) preferentially bind to LXR response elements (LXREs) of target genes in the form of obligate heterodimers^4^. Structurally, LXR*α* consists of an N-terminal ligand-independent activation function domain (AF-1), a DNA-binding domain (DBD) containing two zinc finger regions encoded by a single exon 4, a hinge domain and a ligand-binding domain (LBD) encoded by exons 6–10^1,5^. LBD is in charge of ligand binding, receptor dimerization, nuclear localization, and binding to co-activators or co-repressors and trans-activation^4,5^. Due to the absence of H2 and the contiguity of helices 10 and 11, LBD consists of 10 *α*-helices, of which the C-terminal helix 12 is also called AF-2^6^. Alternative splicing of biological genes is an important mechanism for regulating gene expression and generating protein diversity. So far, a total of five *LXRα* transcripts (*α1*, *α2*, *α3*, *α4* and *α5*) have been identified in human, which are generated by alternative promoter usage and alternative splicing of pre-mRNA^3,7^. The expression levels of *LXRα2*, *LXRα3*, *LXRα4* and *LXRα5* are lower in many human tissues than that of *LXRα1*^3,7^. Two LXR*α* transcripts *LXRα1* and *LXRα2* have been identified in swine, and they differ only in the 5′ untranslated region (UTR)^8^. Among them, *LXRα1* was expressed in various porcine tissues, while *LXRα2* was mainly expressed in thymus and spleen. However, as far as we know, there is no report on the alternative splicing of *LXRα* gene in ruminants.

Like other nuclear receptors, transcripts of *LXRα* have different expression patterns and altered transcriptional activities, and the expression of these transcripts can regulate LXR signaling^3^. In recent years, LXR*α* has been demonstrated to play a crucial role in milk fat synthesis in buffalo and cattle^9,10^. Elucidating the transcriptional and post-transcriptional regulation of buffalo *LXRα* gene in mammary gland is of great significance for understanding the mechanism of buffalo milk fat synthesis. The aim of this study was to identify potential alternative splicing variants of LXR*α* in buffalo mammary gland and investigate the molecular characteristics, expression patterns, and functional roles of different variants. This study will generate fundamental information for exploring the function and regulation of *LXRα* gene in buffalo milk fat synthesis.

## Results

### Identification of alternative splicing transcripts of buffalo *LXRα*

**Figure 1:**
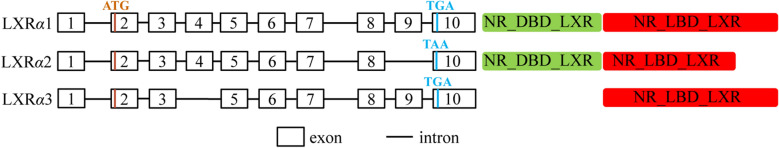
Schematic diagram of three splicing modes and translation termination sites of buffalo *LXRα* transcription products. The left panel depicts the exon/intron organization and structure of the three buffalo *LXRα* transcripts. The right panel displays the structural domains of the LXR*α* variants. Alternative splicing involving skipping of exon 9 or exon 4 generates the transcripts *LXRα*2 and *LXRα*3, respectively.

**Figure 2:**
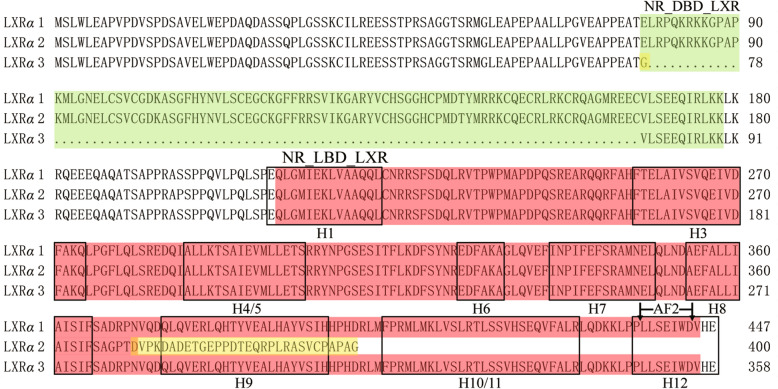
Alignment and structural characteristics of buffalo LXR*α* variants. The green and red shades denote the DNA-binding domain (NR_DBD_LXR) and ligand-binding domain (NR_LBD_LXR), respectively. The yellow shades represent the mismatched amino acids. Boxes indicate helices (H) in the ligand-binding domain (LBD). The region between the arrows represents the AF-2 region. *LXRα*2 is produced by the splicing removal of exon 9 resulting in a truncation of 47 amino acid residues at the C-terminus. *LXRα*3 is generated by the skipping of exon 4, leading to an in-frame deletion of 89 amino acid residues in the DNA-binding domain (DBD).

Through 5′ RACE-PCR, only one type of 5′ UTR sequence of *LXRα* gene was obtained, and further BLAST analysis showed that it was completely consistent with the published 5′ UTR sequence of buffalo *LXRα* with accession number XM_025266088. Further, thirty clones with recombinant LXR*α*-CDS-pMD18-T vectors were randomly selected for sequencing. Three alternative splicing transcripts of buffalo *LXRα* were obtained which were identical to the predicted transcripts reported in NCBI database. The CDS identical to XM_025266088 (1344 bp; predicted transcript variant X1) was referred as buffalo transcript *LXRα1*, identical to XM_025266090 (1203 bp; predicted transcript variant X2) as buffalo transcript *LXRα2*, and identical to XM_025266091 (1077 bp; predicted transcript variant X3) as buffalo transcript *LXRα3*. The CDS sequence consistency of the same type of *LXRα* transcripts between buffalo and cattle was higher than 98.51%. The CDSs of all three *LXRα* transcripts were submitted to GenBank with the accession numbers MZ927742, MZ927743 and MZ927744. The *LXRα2* is generated by the deletion of exon 9, whereas *LXRα3* is by absence of exon 4 (Figs. 1 and 2).

**Figure 3:**
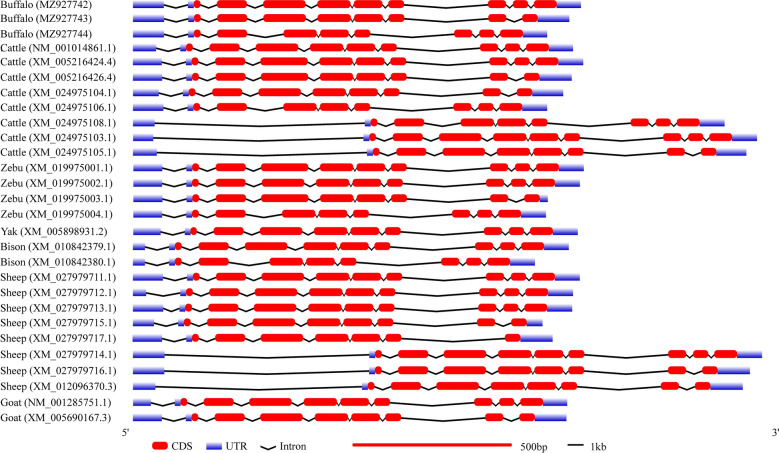
Transcriptional region structure of *LXRα* in some Bovidae species.

In order to have a deeper understanding of the structure of the transcriptional region of *LXRα* gene in buffalo and the alternative splicing mode of the transcript, the structure of the transcriptional region of the transcript obtained in this study was compared with that of all the transcripts of *LXRα* gene of other Bovidae species. Buffalo and other Bovidae species have similar alternative splicing transcripts of *LXRα*. There are three main types of transcripts of this gene in Bovidae species, that is, in addition to the type containing all 10 exons, there are also deleted types of exon 4 or exon 9 (Fig. 3). In addition, there is a transcript in which exons 8 and 9 are removed together in sheep (XM_027979717.1). It is worth noting that the transcripts with the same splicing pattern in Bovidae species have essentially the same CDS length, but their UTR and intron lengths are inconsistent.

### Characteristics, structures and functions of buffalo LXR*α* variants

The molecular characteristics and functions of LXR*α* variants encoded by the alternative splicing transcripts of buffalo *LXRα* were predicted using bioinformatics methods and shown in Table 1. All the variants of buffalo LXR*α* are hydrophilic proteins, distributed in the nucleus with high scores. The molecular functions and biological processes of the LXR*α*1 and LXR*α*2 are the same. Both of them are involved in lipid metabolism and transcriptional regulation. However, compared with the LXR*α*1 and LXR*α*2, the LXR*α*3 loses its DNA-binding activity as a transcription factor and could not participate in transcriptional regulation.

**Table 1:** Characteristics and functions of three variants of buffalo LXR*α*.

	LXR*α*1	LXR*α*2	LXR*α*3
Amino acids	447	400	358
Isoelectric point (pI)	6.62	5.69	5.13
Grand average of hydropathicity	− 0.413	− 0.475	− 0.321
Subcellular localization	Nuclear (10.0)	Nuclear (9.9)	Nuclear (9.8)
Biological process	Lipid metabolic process and regulation of transcription	Lipid metabolic process and regulation of transcription	Lipid metabolic process
Molecular function	Nuclear receptor activity, DNA binding, zinc ion binding, sequence-specific DNA binding and DNA-binding transcription factor activity	Nuclear receptor activity, DNA binding, zinc ion binding, sequence-specific DNA binding and DNA-binding transcription factor activity	Nuclear receptor activity and DNA binding

The completed LXR*α* (LXR*α*1) contains a DNA-binding domain (NR_DBD_LXR) and a ligand-binding domain (NR_LBD_LXR). The NR_LBD_LXR domain is composed of 10 helices and the last helix is the AF-2 (Fig. 2). Because the length of exon 9 is 95 bp, its deletion leads to a change in the reading frame of LXR*α*2 and a mismatch of 30 amino acid residues at the C-terminus. The introduction of the stop codon (TAA) leads to the truncation of 47 amino acids at the C terminus of LXR*α*2, resulting in the deletion of helix 9 to helix 12 in its LBD, which contains AF-2. The skipping of exon 4 in this gene leads to the loss of DBD composed of 89 amino acids, which leads to the transcript *LXRα3*, but this does not change the reading frame (Fig. 2).

### Phylogenetic and sequence identities analysis

**Figure 4:**
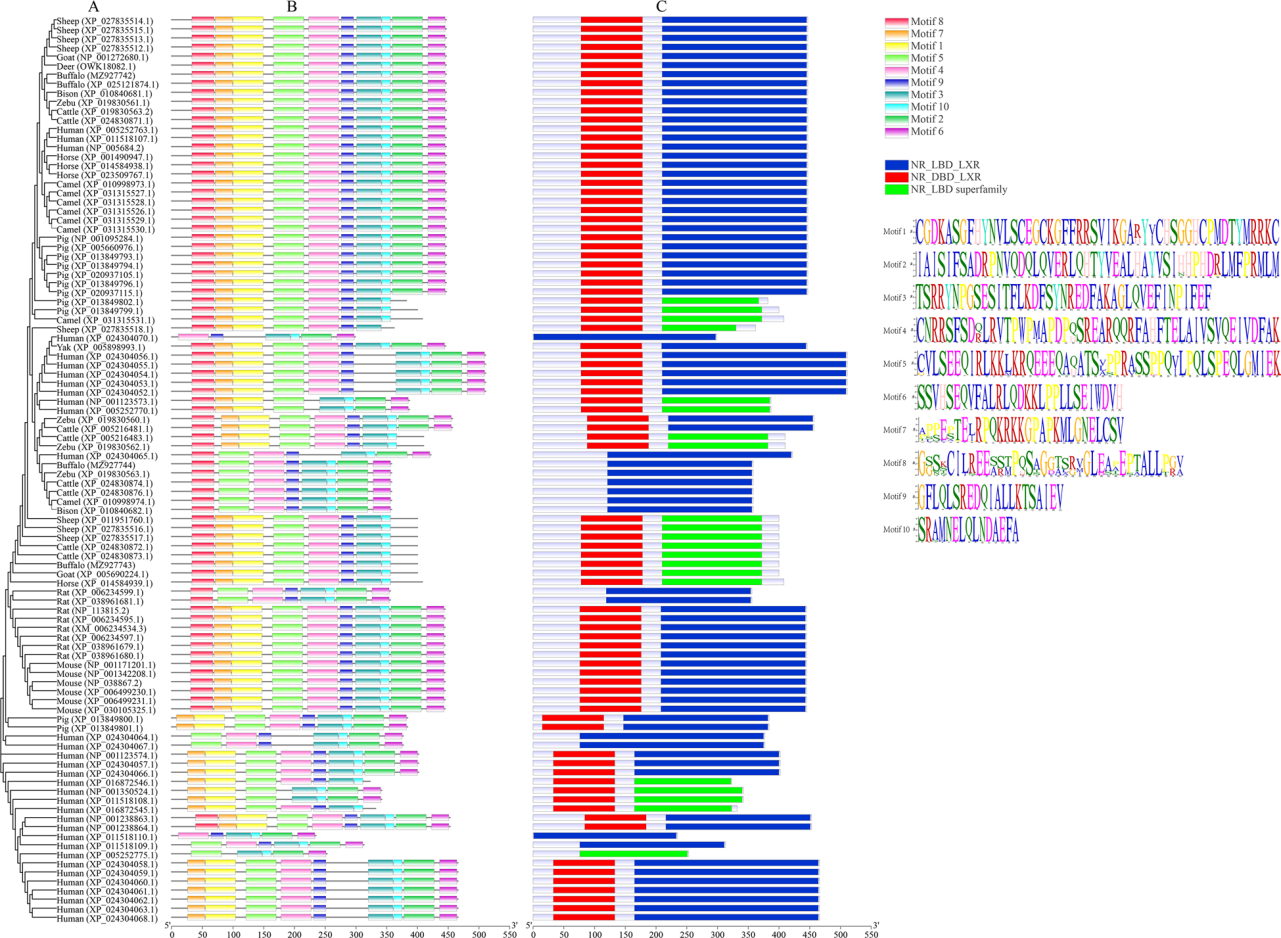
Phylogenetic relationships, motifs and conserved domains of LXR*α* in 14 mammalian species. (**A**) phylogenetic tree; (**B**) motif pattern; (**C**) conserved domain. The conserved motifs and domains in LXR*α* were marked with different color boxes.

The phylogenetic relationship and comparison of motifs and conserved domains between buffalo and 13 representative mammalian LXR*α* variants are illustrated in Fig. 4. In the phylogenetic tree, the variants with the same splicing pattern were clustered into one branch, and the buffalo showed a closer genetic relationship with other species of Bovidae (Fig. 4A). A total of 10 conserved motifs were found in LXR*α* variants across 14 representative mammals (Fig. 4B). Among them, motifs 3 and 10 are found in variants of all species. The protein encoded by the buffalo transcript with accession number MZ927743 lacks motifs 2 and 6 (skipping of exon 9), while the protein encoded by the buffalo transcript with accession number MZ927744 lacks motifs 1 and 7 (skipping of exon 4). In other species, variants with the same splicing pattern as buffalo contain the same motif pattern. In contrast, the motif pattern of LXR*α* variants in human is more complicated. As far as conserved domains are concerned, mammalian LXR*α* variants contain either two complete domains of DBD and LBD, one complete domain of DBD and part of LBD, or only one LBD domain or part of LBD (Fig. 4C). In all mammals, the LBD domain is incomplete due to exon 9 skipping, but it still belongs to the NR_LBD superfamily domain, while the variant with exon 4 skipping contains only LBD domain. The results here indicate that the motifs and conserved domains of mammalian LXR*α* variants are highly consistent.

### Subcellular localization of buffalo LXR*α* variants

**Figure 5:**
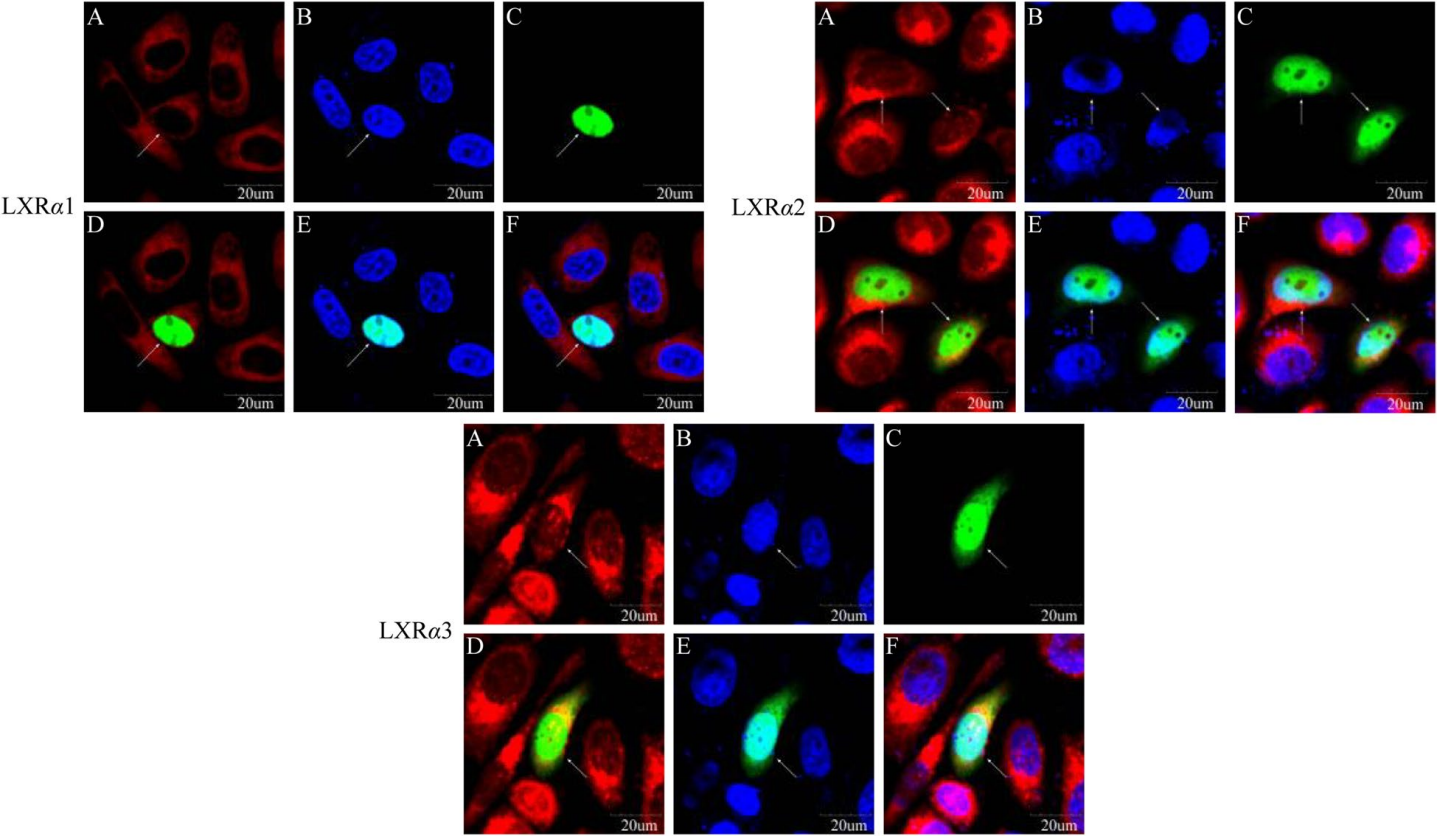
Subcellular localization of three variants of buffalo LXR*α* in BuMECs. (**A**) Mitochondria stained by Mito Tracker; (**B**) nucleus stained by Hoechst 33342; (**C**) green fluorescent protein (GFP); (**D**) merged overlaid GFP and mitochondria; (**E**) merged overlaid with GFP and nucleus; (**F**) merged overlaid GFP, mitochondria and nucleus.

Subcellular localization experiments were carried out to determine the distribution of the three *LXRα* variants in BuMECs. The recombinant vectors of pEGFP-LXR*α* encoding the three *LXRα* variants of buffalo were constructed and transfected into BuMECs respectively. The results of transfection with pEGFP-LXR*α*1 revealed that all the green fluorescent protein (GFP) overlapped with the blue fluorescence of the nucleus, but did not coincide with the red fluorescence of mitochondria (Fig. 5). Although most of the GFP of pEGFP-LXR*α*2 and pEGFP-LXR*α*3 coincided with the blue fluorescence of the nucleus, some of the GFP overlapped with the red fluorescence of the mitochondria. More results of positive cells are shown in Supplementary Fig. S1. The above results indicate that buffalo LXR*α*1 protein functions in the nucleus, while the LXR*α*2 and LXR*α*3 proteins are mainly distributed in the nucleus of BuMECs, but some are also distributed in the cytoplasm.

### Expression of buffalo *LXRα* in mammary tissue

**Figure 6:**
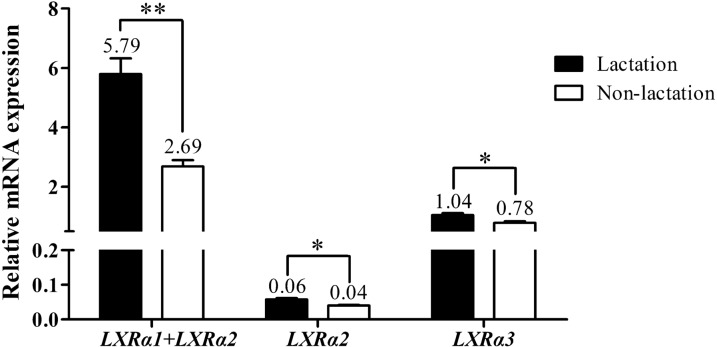
Expression of buffalo *LXRα* transcripts in mammary gland tissue. Data are presented as means ± SEM; *P < 0.05, **P < 0.01.

To investigate the role of *LXRα* in lactation, the mRNA expression differences of the three splicing transcripts of buffalo *LXRα* in the mammary glands between lactation and non-lactation were analyzed (Fig. 6). Although the expression of *LXRα1* alone could not be detected in this study, the expression of *LXRα1* and *LXRα2* together, and the expression of *LXRα2* alone could be detected. Therefore, the expression level of *LXRα1* in mammary gland can be inferred. Compared with non-lactating stage, the mRNA abundance of the three splicing transcripts was significantly increased in lactating stage (*P* < 0.05) (Fig. 6). Furthermore, the relative mRNA abundance of *LXRα2* was the lowest in the mammary gland of the two stages, while the expression of *LXRα1* in the two stages was the highest. The transcript with the highest expression of the same gene is the major transcript^11^. Therefore, *LXRα1* is identified as the major transcript expressed in buffalo mammary gland.

## Discussion

Pre-messenger RNA splicing is an essential process required for the expression of most genes in eukaryotic cells, which provides an important way to regulate gene expression^12,13^. Alternative splicing is the process of producing different mRNA splicing isoforms through different splicing methods in a mRNA precursor, which can cause the final protein product to exhibit different or mutually antagonistic structural and functional properties^14,15^. It can increase the diversity of mRNAs expressed from the genome and is a source of increase in genomic expression and organism complexity^16,17^. As a core element of gene regulation, it is involved in almost all biological processes, such as cell proliferation, growth, differentiation, metabolism, apoptosis and signal transduction^18,19^, and previous studies have shown that it is also related to lactation^20,21^. From the perspective of protein products, there are two main types of alternative splicing: one is the splicing that leads to insertion or deletion, and the other is the splicing that leads to exon substitutions^22^. Notably, RNA editing is also closely related to alternative splicing. RNA editing and alternative splicing synergistically regulate gene transcription^23^, while SNPs associated with RNA editing are also able to alter the translation efficiency of transcripts^24^. In this study, three variants of LXR*α* were identified in buffalo mammary glands. Among them, the variants LXR*α*2 and LXR*α*3 are derived from variant LXR*α*1 via the skipping of exon 9 and exon 4, respectively. It is worth noting that the skipping of exon 4 in LXR*α*3 does not cause a shift in the reading frame. Exons that do not cause frame-shifting when removed are called cassette exons, and most eukaryotic genes have evolved such exons^25^. In the present study, deletion of exon 9 resulted in a preceding stop codon (TAA) in the ORF of the *LXRα2* transcript, resulting in a truncated protein terminated at the 400th amino acid residue. Comparative analysis showed that the structure of the *LXRα* transcripts of buffalo and cattle was highly similar, and the corresponding *LXRα* CDSs of buffalo and cattle were highly consistent. Phylogenetic analysis also showed that buffalo had close genetic relationship with other Bovidae species. In addition, the motifs and conserved domains of buffalo LXR*α* protein are highly consistent with those of other mammal species. These results suggest that buffalo LXR*α* may have similar functions with other mammals, especially Bovidae species, indicating that the *LXRα* gene is functionally conserved in mammals.

LXR*α* is ligand-dependent transcription factor and plays a crucial role in the regulation of cholesterol homeostasis, fatty acid biosynthesis and glucose metabolism^8^. LXR*α* binds with RXR to form a heterodimer, which trans-activates some of lipogenic target genes of LXR*α*^5^. During lactation, the mammary gland is one of the most active metabolic organs with high short-term lipid synthesis capacity^26^. In this study, all three *LXRα* transcripts were expressed in buffalo mammary gland, indicating that the buffalo RXR-LXR*α* heterodimer regulates the transcription of multiple lipogenesis-related downstream genes in the buffalo mammary gland. LXR*α* has been shown to promote lipid synthesis in mammary gland by regulating the expression of multiple genes during the process of milk fat synthesis in buffalo and cattle, especially through the regulation of LXRE site in the core promoter region of its target genes^10,27^. Consistent with previous reports of increased expression levels of *LXRα* in murine mammary epithelial cells and bovine mammary gland tissues from non-lactation to lactation^28,29^, this study showed that the mRNA abundance of the three *LXRα* transcripts of buffalo in lactation stage were all significantly higher than those in the non-lactation period, indicating that LXR*α* is an important regulator of milk fat synthesis, which is also consistent with previous finding^10^. Notably, although there was a significant difference in the expression of *LXRα2* and *LXRα3* between lactation and non-lactation, the magnitude of the difference is very small. Compared with the transcripts *LXRα2* and *LXRα3*, the expression of *LXRα1* in buffalo mammary gland was higher during lactation and non-lactation, and there was an extremely significant difference in expression between the two periods. Therefore, we speculate that the transcript *LXRα1* plays a major regulatory role in the mammary gland of buffalo.

It was confirmed that *LXRα1* was the main transcript in the mammary gland of buffalo by expression analysis. It contains all 10 exons with CDS length of 1344 bp and encodes a precursor of 447 amino acid residues, which corresponds to the human LXRα1 protein^3^. Buffalo LXR*α*1 contains two complete functional domains, namely NR_DBD_LXR (DBD) and NR_LBD_LXR (LBD). Previous studies have shown that the domain DBD is composed of two highly conserved C4-type zinc fingers that confer the ability to bind DNA^5^. The LBD domain of LXR*α* has a dimerization interface and a ligand-dependent AF-2 region at the carboxyl terminus^30^. Upon binding of ligand to the LBD, a conformational change leads to recruitment of co-factors and transcriptional activation^31^. In addition, 10 *α*-helices in the LBD play an important role in the overall topography of LBD^6^. When the agonist binds to the core of the LBD, the conformational change involving helix 12 occurs, and the helix 12 forms a groove with the helices 3 and 4 to introduce the binding site of the co-activators^6^. Deletion of the 9–12 helix at the carboxyl terminus of LXR*α*2 (skipping exon 9) results in an incomplete LBD domain, which may affect its binding to the ligand and lead to a decrease in the ability of transcriptional activation of target genes. Surprisingly, this study revealed that LXR*α*2 and LXR*α*1 may have the same molecular functions and participate in the same biological processes. Because LXR*α*2 has a complete DBD, but does not have a complete LBD domain, it is speculated that LXR*α*2 may compete with LXR*α*1 at the target gene DNA binding site, and thus interfere with the transcriptional activation process in which LXR*α*1 participates. This suggests that the principal function of exon 9 skipping may be the regulation of transcriptional regulation involving LXR*α*1. Furthermore, previous study has shown that RXR mediates nuclear import of RXR-LXR*α* isoforms^32^, and deletion of helices 9 and 10 affects the relative stability of RXR-LXR*α*2 heterodimers, which may result in the inability of LXR*α*2 to fully transfer to the nucleus to perform its functions. This was reinforced by subcellular localization analysis of LXR*α*2 in this study. However, due to the low expression of LXR*α*2 in the mammary gland, the extent of its effect on LXR*α*1 is likely to be small. As for LXR*α*3, skipping of exon 4, which encodes the DBD domain, causes it to lose its ability to bind to target gene promoters. This DBD-free variant may be inactive, but because it possesses an intact LBD domain, it may compete with LXR*α*1 for ligand binding, thereby antagonizing the transcriptional activation activity of LXR*α*1. Interestingly, LXR*α*3 still has partial DNA binding function, which may be due to the ability of N-terminal AF-1 to coordinate gene expression with AF-2^33^. Indeed, the loss of DBD may also lead to the cytoplasmic localization of certain LXR*α*3, as does FOXP2^34^, which was also confirmed by the subcellular localization of LXR*α*3 in this study. In conclusion, three LXR*α* variants of buffalo have different functions in the mammary gland. Among them, LXR*α*1 may play a key role in the transcriptional regulation of target genes, while LXR*α*2 and LXR*α*3 may have an antagonistic regulatory relationship with LXR*α*1. Whether this is the case for the functions inferred here for LXR*α*2 and LXR*α*3 requires further experimental verification.

Alternative splicing events may also accelerate paths of evolution^25^. If the rapidly evolving exon is endowed with a useful function, and the new function has only local benefits, it can make the splice sites tissue-specific^35^. Therefore, alternative splicing contributes to the tissue-specific function of the gene. Tissue-specific alternative splicing may be due to the tissue-specific expression of splicing factors and the corresponding regulation of target mRNA transcripts^13^. A previous study reported the existence of a variant of “LXR*α*3” in humans with a deletion of 60 amino acids in exon 6^3^, but we did not identify this transcript in the buffalo mammary gland. Based on the fact that this “LXR*α*3” was identified in human embryonic kidney cells, we believe that the variant may not exist in the mammary gland and is not associated with lactation. This also gives us reason to believe that exon 6 is essential for the function of LXR*α* in lactation. Exon 6 may have been acquired by alternative splicing during the evolution of mammalian mammary glands. Whether this is the case requires further research.

## Materials and methods

### Ethics declarations

All experiments involving the use of animals were approved by the Experimental Animal Use and Ethics Committee of Yunnan Agricultural University under Contract No. YNAU2019llwyh019. All methods were performed in accordance with the relevant guidelines and regulations and the study is reported in accordance with ARRIVE guidelines.

### Sampling, RNA extraction, and cDNA synthesis

Ten female healthy Binglangjiang buffalo (approximately 4-year-old, river type buffalo) were selected for the sampling of expression analysis in Tengchong city, Yunnan, China. The samples of mammary gland tissues from five lactating buffalo (60 d postpartum) and five non-lactating buffalo (60 d before parturition) were obtained surgically as previously described^36^, and immediately stored in liquid nitrogen for subsequent RNA extraction. Total RNA was extracted from the mammary gland tissue according to manufacturer’s instructions for Trizol reagent (Invitrogen, USA). The quantity and quality of RNA extracted were determined by the NanoDrop 2000UV–Vis spectrophotometer (Thermo Fisher Scientific, Waltham, MA, USA) and electrophoresis in 1.5% denatured agarose gel electrophoresis. The cDNA was synthesized from 2 μg of purified total RNA using a RT reagent Kit with gDNA Eraser (TaKaRa, China) and SMARTer^®^ RACE 5′/3′ Kit (TaKaRa).

### Isolation of 5′ UTR of buffalo *LXRα* gene

The 5′ UTR sequence of the *LXRα* gene was obtained based on the Rapid amplification of cDNA ends (RACE) technique using SMARTer^®^ RACE 5′/3′ Kit following the protocol of the manufacturer. The gene specific primer (GSP) and nested antisense primer (NGSP) were designed on the basis of intermediate known sequences of buffalo *LXRα* (XM_025266088, XM_025266090 and XM_025266091) (Table 2). The 5′ RACE PCR was performed in a 50 μL reaction mixture contained 2 μL 5′ RACE-Ready cDNA, 5 μL of 10× UPM, 1 μL of GSP (10 μM), 16 μL of PCR-Grade H_2_O, 25 μL of 2× SeqAmp Buffer and 1 μL of SeqAmp DNA Polymerase. A touchdown PCR program was employed as follows: (A) 5 cycles of 94 °C for 30 s and 72 °C for 2 min, then (B) 5 cycles of 94 °C for 30 s, 70 °C for 30 s and 72 °C for 2 min, following (C) 25 cycles of 94 °C for 30 s, 68 °C for 30 s and 72 °C for 2 min. Further, a nested PCR was carried out by the UPM short (supplied in kit) and NGSP (10 μM). PCR product was detected on 1.5% agarose gel and purified using Gel Extraction Kit (OMEGA, USA). The purified products were inserted into the pMD18-T vector (Takara) and sequenced bidirectionally by Shanghai Biological Engineering Technology Services Co., Ltd (Shanghai, China).

**Table 2:** Primer information for cloning, subcellular localization and qPCR analysis.

Primer name	Primer sequences (5′–3′)	Product length (bp)	Annealing temperature (°C)	Purpose	Efficiency
GSP	TTTTCCGCTTTTGTGGACGAA		68	5′ RACE	
NGSP	TGTGCTGGATTCCTCCCTGA		68		
LXR*α*_F1	GGTTTAAGAGTGGCCCTGACATCACA	1601	61.6	Gene cloning	
LXR*α*_R1	CTTTAATGCCACGTGAGGATCTCCC				
LXR*α*_F2	ctcgagATGTCTTTGTGGCTGGAGGC	1344/1203/1077	60.1	Subcellular localization	
LXR*α*_R2	gaattcGTTCGTGCACATCCCAGATCT				
LXR*α*1 + 2_qF	CCAGAGGCCACAGAGCTTCGTC	289	62.4	LXR*α*1 + LXR*α*2	1.90
LXR*α*1 + 2_qR	ATAAGACACACTCCTCCCGCAT				
LXR*α*2_qF	AGGCCACAGGTGTCTTATCAGAA	225	60.1	LXR*α*2	1.91
LXR*α*2_qR	GCCAAGGCGTGACTCGAAGC				
LXR*α*3_qF	CCAGATCGCCCTGCTGAAGACG	269	59.1	LXR*α*3	1.87
LXR*α*3_qR	AACATCAGTCGGTCCTGCTG				
ACTB_qF	TGGGCATGGAATCCTG	196	60	ACTB	1.94
ACTB_qR	GGCGCGATGATCTTGAT				
GAPDH_qF	ATGGAGAAGGCTGGGGCTCA	144	60	GAPDH	1.92
GAPDH_qR	GCAGGAGGCATTGCTGACAA				
RPS23_qF	ACCGACGAGACCAGAAGT	306	60	RPS23	1.91
RPS23_qR	CTCCAGGAATGTCACCAA				

The lowercase letters are the restriction sites of *Xho*I and *EcoR*I.

### Cloning of buffalo *LXRα* CDS

**Figure 7:**
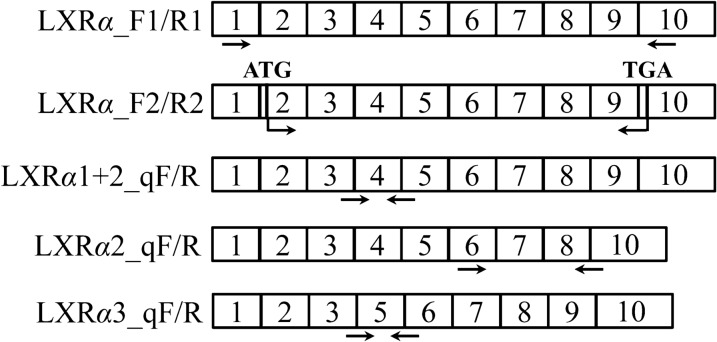
Schematic locations of CDS cloning, subcellular localization and qPCR primers of *LXRα* in this study. Primer pairs of LXR*α*_F1/R1 were used to clone buffalo *LXRα* CDS. LXR*α*_F2/R2 were designed to identify the subcellular localization of LXR*α*1, LXR*α*2 and LXR*α*3. LXR*α*1 + 2_qF/R were designed to detect the relative expression of *LXRα1* + *LXRα2*. LXR*α*2_qF/R and LXR*α*3_qF/R were utilized to detect the relative expression of *LXRα2* and *LXRα3*, respectively.

A pair of primers for cloning the complete CDS of buffalo *LXRα* were designed according to the obtained 5′ UTR sequence in this study and middle known sequence of buffalo *LXRα* (accession no. XM_025266088) (Table 2 and Fig. 7). The cDNA producing from mammary gland was invoked as the template for PCR reaction, and the reaction mixture and protocol were performed in agreement with the manufacturer’s protocols of 2× PCR Master Mix (CWBIO, China). The target PCR products were purified and then introduced into the pMD18-T vector. At least thirty independent clones were sequenced.

### Identification and characterization of buffalo *LXRα* transcripts

The raw sequencing data were assembled, checked and edited by programs SeqMan and EditSeq (DNAstar Inc., USA) to obtain transcript sequences. The open reading frame (ORF) of each transcript and its corresponding amino acid sequence were determined by EditSeq. The gene was identified by homologous search using the BLAST program (http://www.ncbi.nlm.nih.gov/Blast.cgi). The sequence of buffalo *LXRα* gene (NC_059172.1) was downloaded and the exon sequences of this gene were obtained according to the gene structure annotation information. The alternative splicing pattern of this gene was determined by comparing the exon sequences of this gene with the transcripts obtained in this study. Physicochemical characteristics and subcellular localization of LXR*α* variants were predicted by the ProtParam tool (https://web.expasy.org/protparam/) and ProtComp 9.0 (http://linux1.softberry.com/berry.phtml). Biological process and molecular function analysis were conducted using InterProScan (http://www.ebi.ac.uk/interpro/search/sequence-search). Furthermore, a phylogenetic tree was constructed using the Mega 6 based on the amino acid sequences using the maximum likelihood method^37^.

The genome annotation GTF file of buffalo, cattle, zebu, bison, sheep and goat was downloaded from NCBI Datasets (https://www.ncbi.nlm.nih.gov/datasets/) to obtain all the transcription information of *LXRα* gene. Genetic structure information was added to the *LXRα* transcripts of these species using the GXF Fix function of TBtools software^38^. Further, all the gene structure information of the transcripts was submitted to the Gene Structure Display Server 2.0 (http://gsds.gao-lab.org/) for gene structure visualization. Conserved motifs of LXR*α* proteins were analyzed by website MEME (http://meme-suite.org/tools/meme). Conservative domains were determined using NCBI Batch Web CD-Serach Tool (https://www.ncbi. nlm.nih.gov/Structure/bwrpsb/bwrpsb.cgi).

### The quantitative real-time PCR (qPCR)

Specific qPCR primers were designed to measure the relative expression of three *LXRα* transcripts according to the obtained CDS sequences of buffalo *LXRα* in this study (Table 2 and Fig. 7). The geometric mean of the Ct values of *β*-actin (*ACTB*), glyceraldehyde 3-phosphate dehydrogenase (*GAPDH*) and ribosomal protein S23 (*RPS23*) genes was utilized to normalize the expression of target mRNA. The primer pair LXR*α*1 + 2_qF/R were designed to detect the relative expression of both *LXRα1* and *LXRα2* (there was no suitable primer pair to detect the expression of *LXRα1* alone), and LXR*α*2_qF/R and LXR*α*3_qF/R were used to detect the relative expression of *LXRα2* and *LXRα3*, respectively. The qPCR was performed on a CFX96 Real-Time System (Bio-Rad, Hercules, CA) with TB Green^®^ Advantage^®^ qPCR Premix (TaKaRa) under manufacturer’s protocols. The qPCR analyses of each transcript in this study were performed using cDNA from 5 biological replicates, with 3 technical replicates per biological replicate.

### Cell culture

Buffalo mammary epithelial cells (BuMECs) were obtained from a lactating buffalo (60 d postpartum; 5-year-old) and purified based on the differential sensitivity of the cells to trypsin digestion as previously described by our lab^39,40^. Dulbecco modified Eagle medium (Gibco, USA) supplemented with 5 μg/mL insulin (Sigma, USA), 5 μg/mL hydrocortisone (Sigma), 1 μg/mL epidermal growth factor (Sigma), 2% penicillin/streptomycin (Gibco) and 10% fetal bovine serum (Gibco) were used for the culture of purified BuMECs. BuMECs were cultured under constant conditions of 37 °C and 5% CO_2_, and the medium was altered every 24 h.

### Subcellular localization of buffalo LXR*α*

The CDSs of three buffalo *LXRα* transcripts that did not contain the stop codon were amplified via PCR. The pEGFP-N1 vector (CLONTECH Laboratories, Inc.) was constructed to express buffalo *LXRα* gene. The CDS of *LXRα* was subcloned into the vector via *Xho*I and *EcoR*I sites to generate pEGFP-LXR*α* recombinant. The details about the primers are presented in Table 2. When the confluence of BuMECs in the 6-well cell culture plates was about 80%, 3 μg of pEGFP-LXR*α* was transiently transfected into the cells according to the instructions of FuGENE^®^ 6 transfection reagent (Invitrogen, USA). After transfection for 48 h, the medium was removed and the cells were washed twice with PBS. After adding Mito-Tracker Red CMXRos (Beyotime, Shanghai, China) with a final concentration of 200 nM, the cells were incubated for 20 min at 37 °C for mitochondrial staining. After the staining solution was removed, the cells were washed twice with PBS (Gibco), Hoechst 33342 (Beyotime) cell nucleus staining solution was added, and further incubated for 20 min at 37 °C. After the staining solution was removed, the cellular localization of LXR*α* protein was observed by confocal laser scanning microscope (OLYMPUS, Japan).

### Data analysis

The qPCR data obtained were displayed with means ± SEM. Data of qPCR were calculated by the 2^−ΔΔCt^ method. Statistical comparison of the means between the two groups was performed using Student’s *t*-test by software GraphPad Prism 5, and *P* value < 0.05 were considered as significant.

## Conclusions

We report for the first time the existence of three transcripts of the *LXRα* gene in buffalo mammary gland, which are derived from alternative splicing of the exons of a single gene. Among these three transcripts, *LXRα1* was the dominant transcript with the highest expression in lactating mammary glands and played a functional role in the nucleus. The mRNA expression of the three *LXRα* transcripts in buffalo mammary glands was significantly increased during lactation compared with non-lactation, suggesting that they may play a crucial role in buffalo milk fat synthesis. The function of buffalo LXR*α*1 protein is very similar to that of other species of Bovidae. The other two LXR*α* variants (LXR*α*2 and LXR*α*3) exhibited some differences from LXR*α*1 in protein properties, structure, molecular function and subcellular localization, suggesting that they have different functions in buffalo mammary glands. The LXR*α*1 may play a key role in transcriptional regulation of target genes, while LXR*α*2 and LXR*α*3 may antagonize the transcriptional regulation of LXR*α*1. This study revealed that post-transcriptional processing of *LXRα* gene plays an important role in the lactation of buffalo.

## Supplementary Information

Supplementary Figure S1.

## Data Availability

The sequences of all three LXRα transcripts are available in GenBank with the accession numbers MZ927742, MZ927743 and MZ927744.
