# Supplementary material for: Identification and characterization of alternative splicing variants of buffalo LXRα expressed in mammary gland
Source: Sci Rep. 2022 Jun 22;12:10588. doi: 10.1038/s41598-022-14771-0 (PMC9218113; doi:10.1038/s41598-022-14771-0)
Supplement: Supplementary file 1 — Supplementary Figure S1. [file 41598_2022_14771_MOESM1_ESM.pdf]

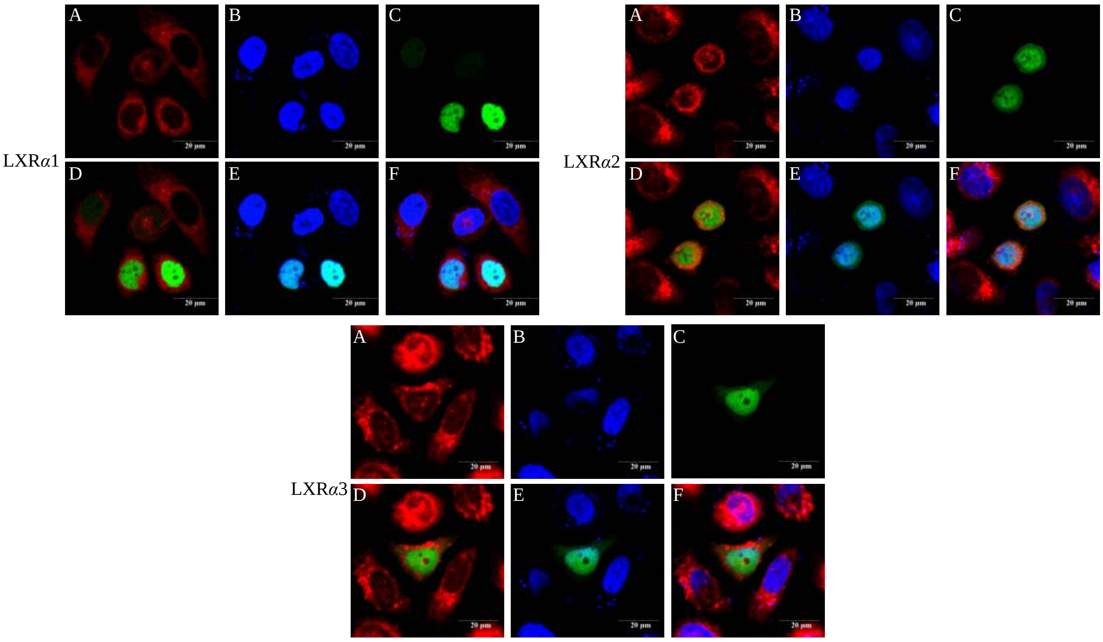

Supplementary Figure S1. Subcellular localization of three variants of buffalo LXR*α* in BuMECs. (A) Mitochondria stained by Mito Tracker; (B) nucleus stained by Hoechst 33342; (C) green fluorescent protein (GFP); (D) merged overlaid GFP and mitochondria; (E) merged overlaid with GFP and nucleus; (F) merged overlaid GFP, mitochondria and nucleus.
